# Anti-Obesity Effect of *Lactobacillus gasseri* BNR17 in High-Sucrose Diet-Induced Obese Mice

**DOI:** 10.1371/journal.pone.0054617

**Published:** 2013-01-30

**Authors:** Ji-Hee Kang, Sung-Il Yun, Mi-Hee Park, Jun-Hong Park, So-Young Jeong, Han-Oh Park

**Affiliations:** R&D center, Bioneer Corporation, Daejeon, Republic of Korea; University of Bremen, Germany

## Abstract

Previously, we reported that *Lactobacillus gasseri* BNR17 (BNR17), a probiotic strain isolated from human breast milk, inhibited increases in body weight and adipocyte tissue weight in high-sucrose diet-fed Sprague-Dawley (SD) rats and reduced glucose levels in type 2 diabetes mice. In the current study, we conducted further experiments to extend these observations and elucidate the mechanism involved. C57BL/6J mice received a normal diet, high-sucrose diet or high-sucrose diet containing *L. gasseri* BNR17 (10^9^ or 10^10^ CFU) for 10 weeks. The administration of *L. gasseri* BNR17 significantly reduced the body weight and white adipose tissue weight regardless of the dose administered. In BNR17-fed groups, mRNA levels of fatty acid oxidation-related genes (ACO, CPT1, PPARα, PPARδ) were significantly higher and those of fatty acid synthesis-related genes (SREBP-1c, ACC) were lower compared to the high-sucrose-diet group. The expression of GLUT4, main glucose transporter-4, was elevated in BNR17-fed groups. *L. gasseri* BNR17 also reduced the levels of leptin and insulin in serum. These results suggest that the anti-obesity actions of *L. gasseri* BNR17 can be attributed to elevated expression of fatty acid oxidation-related genes and reduced levels of leptin. Additionally, data suggested the anti-diabetes activity of *L. gasseri* BNR17 may be to due elevated GLUT4 and reduced insulin levels.

## Introduction

Obesity is caused by a multiple factors, including genetic, metabolic, behavioral and cultural factors. More specifically, a high fat intake and low energy expenditure are the main causes of obesity, as well as metabolic disorders such as insulin resistance, type 2 diabetes, and cardiovascular diseases [1], [2]. A variety of programs and treatments including drug therapeutics, surgical intervention and dietary control for obesity management or prevention have been developed; however, these are often associated with safety issues. Therefore, the development of a safe and effective dietary supplement to assist with body weight management is essential.

Lactobacilli and bifidobacteria are representative probiotic microorganisms that benefit human health through modulation of the immune system [3], prevention of cancer [4], enhancement of intestinal functions [5] and a hypocholesterolemic effect [6]. Recently, some studies have expended the functionality of probiotics to obesity management. Some probiotics have been demonstrated to have an anti-obesity property by regulating lipid and glucose metabolism [7], [8], producing conjugated linoleic acid [9], [10], reducing the adipocyte size and increasing the number of small adipocytes in white adipose tissue [11], and regulating leptin [12].

We have observed the effects of *L. gasseri* BNR17, a probiotic strain isolated from human breast milk, on the high-sucrose diet-fed SD rat and transgenic *db/db* mouse [13], [14]. In those studies, *L. gasseri* BNR17 suppressed the body weight and fat weight gain, fasting and postprandial blood glucose, and improved oral glucose tolerance. The purpose of the current study was to extend these observations and elucidate the mechanism involved in the anti-obesity activity of *L. gasseri* BNR17. We investigated the impact of *L. gasseri* BNR17 on body weight gain, fat accumulation, and mRNA expression of obesity-related genes in diet-induced obese mice.

## Materials and Methods

### Animals and Experiment

Male C57BL/6J mice (6-week-old, *n* = 8 per group) were obtained from Central Lab Animal Inc. (Seoul, South Korea). All animals were housed in standard plastic cages (two mice per cage), and maintained under a 12-h light-dark cycle at constant temperature and humidity (23±1°C and 55±5%, respectively) with free access to food and water. This study was carried out in accordance with the recommendations in the guide for the care and use of the Animal, Plant and Fisheries Quarantine and Inspection Agency (Republic of Korea). The protocol was approved by the Committee on the Ethics of Animal Experiments of the Bioneer Corporation (AEC-20081229-0004). Following acclimatization for 1 week, the mice were fed a normal diet (ND) (2918C, containing 6.0% fat and 18.5% protein by weight; Koatech Animal Inc., Pyeongtaek, South Korea), or a high-sucrose diet (HSD) (AIN-76A, 5.0% fat, 50.0% sucrose, 15.0% cornstarch and 20.0% protein by weight; Central Lab Animal Inc.), or high-sucrose diet and BNR17 10^9^ CFU (HSD+BNR17(9)) or 10^10^ CFU (HSD+BNR17(10)) for 10 weeks. *L. gasseri* BNR17 was prepared fresh daily and orally administered twice per day. Body weight and food intake were measured once a week. At the end of the 10-week treatment period, mice were killed by cervical dislocation after blood gathering. Liver, spleen, kidney, and adipose tissues (mesenteric, subcutaneous, epididymal, perirenal) were dissected precisely and weighed. An *in vivo* CT analysis (Inveon™, Siemens Medical Solutions USA Inc.) was carried out prior the killing of animals under 1.5–2% isoflurane in O_2_ anesthesia.

### Real-time PCR Analysis

RNA was extracted from ∼0.1 g of tissues using the RNeasy Mini kit (Qiagen) for liver and RNeasy Lipid Tissue Mini kit (Qiagen) for white adipose tissue, according to the manufacturer’s protocols. cDNA was synthesized using the Accupower® Rocketscript™ Cycle RT Premix kit from Bioneer (Daejeon, South Korea). qPCR was performed using an Exicycler (Bioneer) with Accupower® 2× Greenstar qPCR Master Mix (Bioneer). Primer sequences for the targeted mouse genes are listed in Table 1.

**Table 1:** Primer sequences of mouse mRNA.

Target gene	Forward Primer (5′–3′)	Reverse Primer (5′–3′)	Ref.
PPARα	GTACGGTGTGTATGAAGCCATCTT	GCCGTACGCGATCAGCAT	[15]
PPARδ	GCCATATTCCCAGGCTGTC	CAGCACAAGGGTCATCTGTG	
CPT1	GTGACTGGTGGGAGGAATAC	GAGCATCTCCATGGCGTAG	
ACO	GTGCAGCTCAGAGTCTGTCCAA	TACTGCTGCGTCTGAAAATCCA	
UCP3	CCAGAGCATGGTGCCTTCGCT	CTCGTGTCAGCAGCAGTG	
GLUT4	GGAAGGAAAAGGGCTATGCTG	TGAGGAACCGTCCAAGAATGA	
SREBP-1c	ACGGAGCCATGGATTGCACA	AAGGGTGCAGGTGTCACCTT	
ACC	ATGGGCGGAATGGTCTCTTTC	TGGGGACCTTGTCTTCATCAT	
LPL	CCACAGCAGCAAGACCTTC	AGGGCGGCCACAAGTTTG	
FAS	TGCTCCCAGCTGCAGGC	GCCCGGTAGCTCTGGGTGTA	
Adiponectin	GAGATGCAGGTCTTCTTGGTC	GCTCTCCTTTCCTGCCAG	
TNF-α	AAGCCTGTAGCCCACGTCGTA	GGCACCACTAGTTGGTTGTCTTTG	
ANGPTL4	AAAGAGGCTGCCCGAGAT	TCTCCCCAACCTGGAACA	
β-Actin	GAGCGCAAGTACTCTGTGTG	CGGACTCATCGTACTCCTG	

PPARα**,** peroxisome proliferator-activated receptor α; PPARδ, peroxisome proliferator-activated receptor δ; CPT, carnitine palmitoyl-transferase; ACO, acyl CoA oxidase; UCP3, uncoupling proteins3; GLUT4, glucose transporter 4; SREBP-1c, sterol regulatory element-binding protein-1c; ACC, acetyl-CoA carboxylase; FAS, fatty acid synthetase; PPARγ**,** peroxisome proliferator-activated receptor γ; LPL, lipoprotein lipase; TNF-α, tumor necrosis factor-α.

### Biochemical Analyses

Endocrine peptides (ghrelin, GIP, GLP-1, glucagon, insulin, leptin) were determined using a Bio-Plex suspension array system (Luminex, Austin, USA). Metabolic parameters including glucose, total cholesterol, HDL-cholesterol, and LDL-cholesterol levels in serum were analyzed using a Clinical Analyzer 7020 (HITACHI, Tokyo, Japan).

### Measurement of Adipocyte Size

Adipocyte sizes in the mesenteric, subcutaneous, epididymal and perirenal adipose tissue were measured in paraffin-embedded tissue. Briefly, adipocyte tissues were fixed in 10% neutral formalin solution, embedded in paraffin, cut into 4-µm sections, and stained with hematoxylin and eosin. Cell sizes were measured using a DIXI3000 (Leica, Wetzlar, Germany).

### Statistical Analysis

The data were expressed as mean values with their standard errors. Analyses were performed using pairwise *t*-tests and Wilcoxon rank sum tests. Differences were considered to be statistically significant at values of *P*<0.05.

## Results

### *L. gasseri* BNR17 Inhibits High-sucrose Diet-induced Body Weight Gain and Fat Weight Accumulation

**Figure 1:**
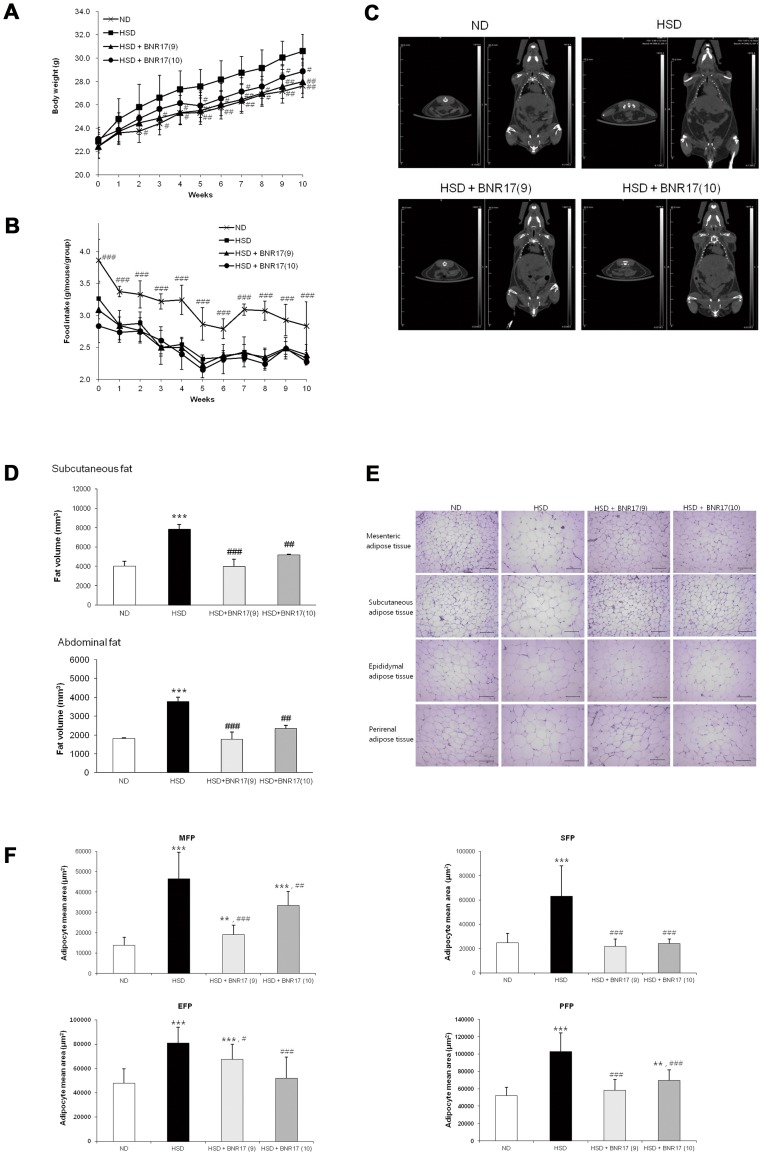
*L. gasseri* BNR17 supplementation decreases high-sucrose diet-induced body weight gain and fat mass accumulation. (A) Change in body weight, (B) change in food intake, (C) representative CT scanning images of abdominal (left) and whole body (right) fat accumulation (in black) at 10 weeks (D) correspond to the volume of subcutaneous and abdominal fat, (E) representative adipose tissue-staining images in mice of four groups, (F) adipocyte mean area (µm^2^). Data represent the means ± SD. Pairwise *t*-test: *P<0.05, **P<0.01, ***P<0.001 *versus* the ND group; ^#^ *P*<0.05, ^##^ *P*<0.01, ^###^ *P*<0.001 *versus* the HSD group.

High-sucrose diet feeding induced significant body weight gain throughout the study period compared to the ND group (Figure 1A and Table 2). The administration of BNR17 induced less body weight gain than the HSD group, although not in a dose-dependent manner. Food intake differed significantly between the ND and HSD groups (Figure 1B and Table 2); whereas no significant differences were observed in daily food intake between the HSD and HSD+BNR17 groups. Moreover, energy intake were similar for all groups. This suggests that BNR17 contributed to the reduced body weight gain. Total cholesterol and LDL-cholesterol in the HSD group and HSD+BNR17 groups increased compared to the ND group; however, no significant reduction was caused by BNR17 administration (Table 2). In addition, glucose levels did not change with BNR17 administration. High-sucrose diet also induced increased adipose tissue weight as compared with normal diet feeding (Table 2). BNR17 administration significantly suppressed the increase of fat mass in all white adipose tissues, including mesenteric, subcutaneous, epididymal and perirenal adipose tissue (Table 2). Further, CT imaging showed a significant reduction in body fat profile with BNR17 treatment (Figures 1C and D). Moreover, HE staining of white adipose tissues revealed that supplementation with BNR17 was associated with a significant reduction in average adipocyte size in mesenteric, subcutaneous, epididymal and perirenal adipose tissues, as compared with the HSD group (Figures 1E and F).

**Table 2:** Body weight, fat weight and organs weight of mice fed the experimental diets for 10 weeks.

	ND	HSD	HSD+BNR17(9)	HSD+BNR17(10)
Initial body weight (g)	22.41±1.06	22.88±1.20	22.44±1.19	22.90±0.77
Final body weight (g)	27.63±1.77	30.59±1.46**	27.98±1.93##	28.35±0.93#
Food intake(g/mouse/day)	3.15±0.20	2.57±0.15***	2.58±0.14***	2.47±0.16***
Energy intake(kcal/mouse/day)	9.75±0.63	9.74±0.56	9.78±0.53	9.36±0.60
Mesenteric fat pad (g)	0.27±0.10	0.44±0.10**	0.29±0.08##	0.37±0.05
Subcutaneous fat pad (g)	0.64±0.10	1.15±0.22***	0.73±0.15###	0.95±0.13**
Epididymal fat pad (g)	0.78±0.17	1.11±0.23**	0.80±0.20##	0.87±0.14
Perirenal fat pad (g)	0.43±0.12	0.65±0.14**	0.47±0.14#	0.55±0.10
Liver weight (g)	1.16±0.13	1.18±0.09	1.01±0.09* ^,^ ##	1.06±0.15
Spleen weight (g)	0.16±0.03	0.18±0.02	0.15±0.02#	0.16±0.02
Kidney weight (g)	0.30±0.02	0.29±0.01	0.28±0.02	0.29±0.02
Cholesterol	140.57±12.88	192.00±24.60**	177.63±19.30**	188.18±18.88**
HDL-cholesterol	69.22±4.91	75.00±4.60	79.28±7.91*	79.32±8.16
LDL-cholesterol	6.19±0.95	18.2±3.40**	16.47±3.44**	18.34±3.20**
Glucose	209.63±30.29	204.00±32.70	200.06±62.73*	214.21±56.52

C57BL/6J mice were fed a normal diet (ND), a high-sucrose diet (HSD) or a HSD containing *L. gasseri* BNR17 (10^9^ or 10^10^ CFU) for 10 weeks. After measurement of body weight and feed intake, the white adipose tissue, liver, spleen and kidney were removed and weighed. Data represent the means ± SD of eight mice per group. Pairwise *t*-test:

* *P*<0.05,

** *P*<0.01,

*** *P*<0.001 *versus* the ND group;

# *P*<0.05,

## *P*<0.01,

### *P*<0.001 *versus* the HSD group.

### *L. gasseri* BNR17 Affects the mRNA Expression of Obesity and Diabetes-related Genes in Liver and White Adipose Tissue

**Figure 2:**
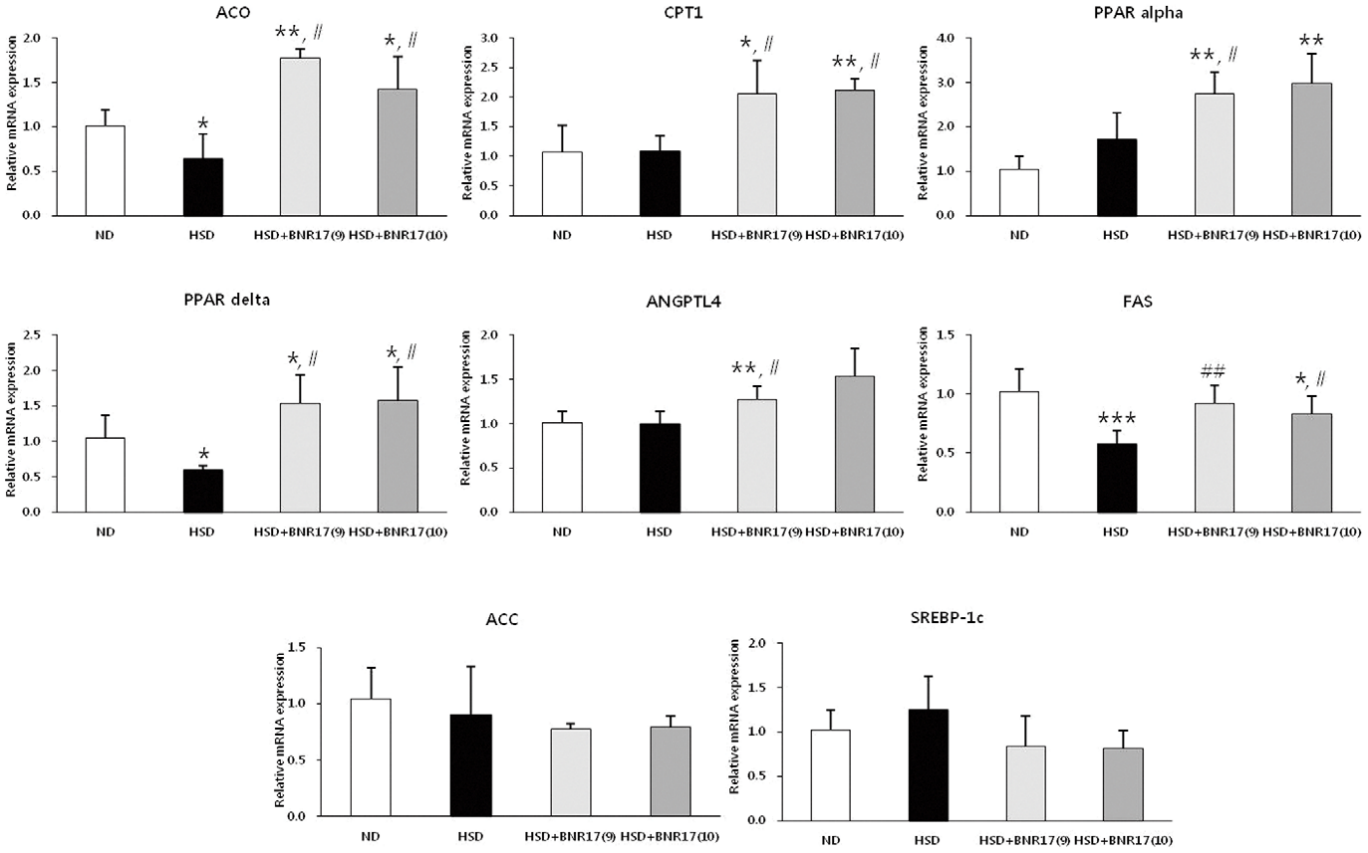
*L. gasseri* BNR17 affects mRNA expression in the liver. C57BL/6J mice were given ND, HSD, or HSD containing BNR17 (10^9^ or 10^10^ CFU) for 10 weeks. The liver was then removed and mRNA expression was measured by real-time RT-PCR using β-actin as a housekeeping gene. Data represent the means ± SD. Pairwise *t*-test: *P<0.05, **P<0.01, *versus* the ND group; ^#^ *P*<0.05, ^##^ *P*<0.01 *versus* the HSD group.

The effect of BNR17 on the expression of obesity-related genes was investigated using real-time RT-PCR. The mRNA expressions of ACO, CPT1, PPARα, PPARδ and ANGPTL4 were significantly higher in the BNR17 groups compared to the HSD group (Figure 2). Furthermore, mRNA expressions of ACC and SREBP-1c showed tendencies to be lower in BNR17 groups.

**Figure 3:**
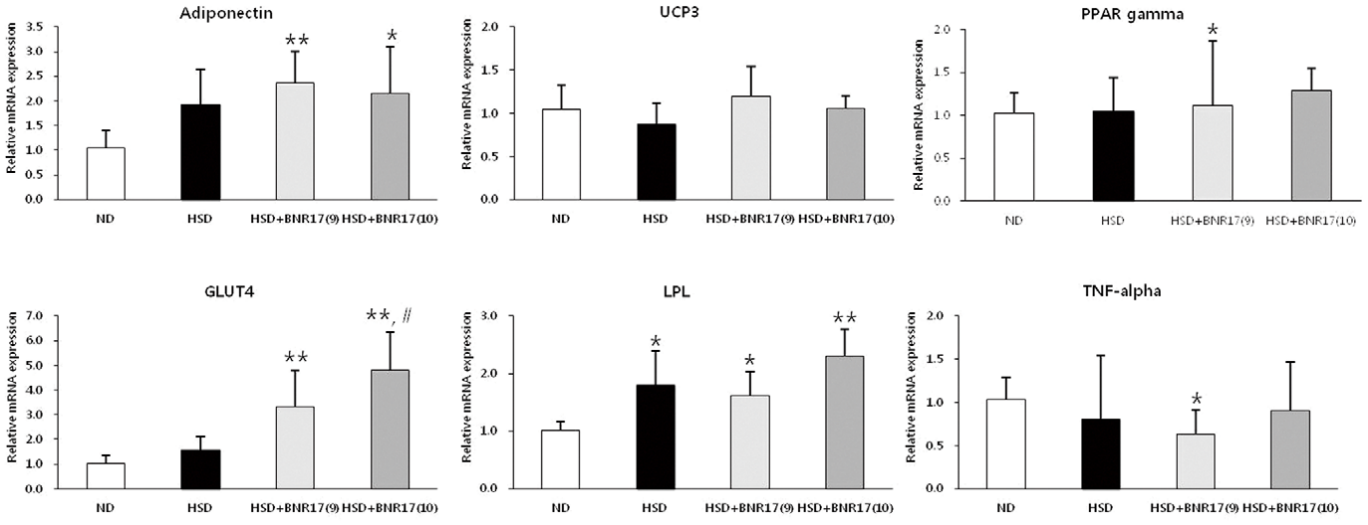
*L. gasseri* BNR17 affects mRNA expression in white adipose tissue. C57BL/6J mice were given ND, HSD, or HSD containing BNR17 (10^9^ or 10^10^ CFU) for 10 weeks. The white adipose tissue was then removed and mRNA expression was measured by real-time RT-PCR using β-actin as a housekeeping gene. Data represent the means ± SD. Pairwise *t*-test: *P<0.05, **P<0.01 *versus* the ND group; ^#^ *P*<0.05 *versus* the HSD group.

The mRNA expressions of adiponectin, UCP3, LPL, PPARγ and TNF-α in white adipose tissue were measured. There were no significant differences between the HSD and BNR17-fed groups (Figure 3). However, the mRNA expression of GLUT4 was higher in the BNR17 groups compared with the HSD group.

### *L. gasseri* BNR17 Reduces the Levels of Leptin and Insulin in Serum

**Figure 4:**
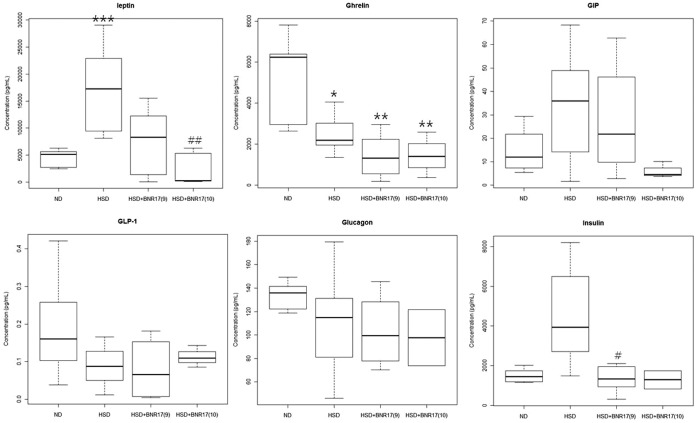
*L. gasseri* BNR17 affects endocrine hormones. C57BL/6J mice were given ND, HSD, or HSD containing BNR17 (10^9^ or 10^10^ CFU) for 10 weeks. Serum was obtained by centrifugation of whole blood and analyzed. GIP, glucose-dependent insulinotropic polypeptide; GLP, glucagon-like peptide. Data represent the means ± SD. Wilcoxon rank-sum test: *P<0.05, **P<0.01, ***P<0.001 *versus* the ND group; ^#^ *P*<0.05, ^##^ *P*<0.01 *versus* the HSD group.

The effect of BNR17 on the gastrointestinal hormones involved in body weight control was investigated. The level of leptin increased in the HSD group compared to the ND group; however it decreased in BNR17-fed groups (Figure 4). Similarly, the level of insulin was significantly lower in BNR17-administered mice. Levels of other hormones among the HSD group and BNR17-fed groups were not different.

## Discussion

Although there was no difference in food and energy intake between the HSD group and BNR17 groups (Figure 1B and Table 2), the increase in body weight was suppressed in the BNR17 groups (Figure 1A and Table 2). There was a significant reduction in subcutaneous and abdominal fat mass in BNR17-fed groups compared to the HSD group (Figure 1C and D). Subcutaneous fat and abdominal fat are the major types of white adipose tissue. Abdominal obesity is associated with increased risk of insulin resistance and cardiovascular diseases, whereas increased subcutaneous fat correlates with a favorable plasma lipid profile [16], [17]. Indeed, the mean adipocyte sizes of all white adipose tissues were remarkably reduced in BNR17-fed mice (Figures 1E and F). Subcutaneous adipocytes are the main source of leptin and adiponectin [16]. Leptin is an adipocyte hormone that controls body weight by regulating food intake and energy expenditure [18], [19]. Leptin concentrations are correlated with the percentage of body fat; higher serum levels have been found in obese individuals compared with non-obese individuals [20]. BNR17 suppressed the elevation of plasma leptin (Figure 3), suggesting that the reductions in fat mass and body weight are associated with a reduction in leptin. Similar effects have been observed in other studies [9], [20], [21]. For the liver, the weight reduction were observed in BNR17 groups (Table 2), however HE staining and O-red staining of liver tissue did not show any changes between groups (Data not shown).

In this study, glucose was not change between groups. In the paper that investigated the role of fatty acid composition in the development of metabolic disorders in sucrose-induced obese rates, the different time courses of the increases in plasma glucose, insulin, and triglycerides during the course of developing obesity suggest that some time- or tissue-dependent process is necessary to induce these metabolic abnormalities [22].

Some studies have reported that the feeding of a low-protein, high-carbohydrate diet (6% protein and 74% carbohydrate) induced an increase in lipid content in the whole carcass, epididymal adipose tissue and retroperitoneal adipose tissue [23]–[25]. Long-term (16 weeks) feeding of a high-sucrose (65%) diet to C57BL/6 mice induced obesity, hepatic steatosis, and insulin resistance [26]. In Asian populations, including Koreans, Chinese and Japanese, the traditional diet is characterized as being high in carbohydrate rather than fat, thus the increasing prevalence of obesity is associated with a high carbohydrate intake. Among Korean adults, a high carbohydrate intake is inversely associated with HDL-cholesterol [27]. In the current study, significant increases in body weight and fat mass in HSD groups were induced for 10 weeks as compared to the normal diet (Figure 1 and Table 2), and increases in lipid profile (total cholesterol, LDL- and HDL-cholesterol) were induced by high-sucrose diet feeding.

Because obesity results from low energy expenditure and increased fatty acid synthesis, we measured the mRNA expression levels of related genes in liver and white adipose tissues. In the liver, the administration of BNR17 significantly increased mRNA expression of ACO, CPT1, ANGPTL4, PPARα and PPARδ, as compared to the HSD group (Figure 2). ACO and CPT1 are considered to be rate-limiting enzymes in mitochondrial fatty acid oxidation [28] and ANGPTL4 is a circulating lipoprotein lipase (LPL) inhibitor that controls triglyceride deposition into adipocytes [29]. These genes are target genes of PPARs, which have essential roles in energy homeostasis and adipogenesis [30], and their expression is increased by the activation and elevation of PPARα and PPARδ, resulting in anti-obesity effects. Excess adipose tissue mass is caused mainly by the differentiation of precursor cells into new adipocytes (adipogenesis). Several transcription factors including CCAAT/enhancer binding protein-α (C/EBPα), PPARγ, SREBP-1c are involved in this process [31]. PPARγ regulates the expression of adipocyte genes such as adipocyte-fatty acid binding protein (A-FABP) [32], and SREBP-1c controls the expression of lipogenic genes such as FAS and ACC [33], [34]. We observed tendencies for reduced SREBP-1c and ACC in the BNR17-fed groups compared to the HSD group; however, we did not detect a reduction in mRNA expression of FAS, the rate-limiting enzyme of fatty acid synthesis in the liver (Figure 2). Moreover, PPARγ and LPL, which are related to fat intake, did not differ among the HSD group and BNR17-fed groups (Figure 3). Therefore, it seems that the anti-obesity effect of BNR17 is responsible for the increased expression of fatty acid metabolism-related genes rather than reduced fatty acid synthesis and fat intake in the liver.

In this study, BNR17 did not show dose-dependent suppression of body weight and fat mass gain as there was no significant difference in biomarkers between the BNR17(9) and BNR17(10) groups. This suggests that BNR17 exhibits anti-obesity activity at doses >10^9^ CFU. This is not consistent with a previous study of the dose-dependent anti-diabetic activity of BNR17 in *db/db* mice [14]. Although we did not clarify the reason in this study, recently, it has been reported that immunomodulation of dendritic cells by probiotics showed very different profiles according to the bacterial inoculum, so the probiotic effect may differ depending on the frequency and size of doses ingested [35]. This means that the determination of the optimal effective dose of probiotics may be required for the future development of commercial products.

Interestingly, we observed changes in several diabetes-related biomarkers in this study. GLUT4 is one of the main glucose transporters expressed in skeletal muscle and adipose tissue. An increase in GLUT4 expression in skeletal muscle is known to ameliorate insulin resistance associated with obesity or diabetes [36], while it has been reported that adipose GLUT4 gene expression changes were more related to insulin resistance and type 2 diabetes rather than obesity [37]. In our study, BNR17 significantly increased GLUT4 mRNA expression in white adipose tissue (Figure 3). Furthermore, the insulin level increased in the HSD group, which was decreased significantly by BNR17 supplementation (Figure 4). In the case of pre-diabetes, increases in blood glucose stimulate the secretion of insulin and subsequently induce hyperinsulinemia with a normal blood glucose range. Hyperinsulinemia is frequently accompanied by obesity, and a biomarker of insulin resistance [38]. It is expected that the regulation of GLUT4 and insulin can likely be attributed to the anti-diabetes activity of BNR17.

Recently, many studies have reported the preventive activity of probiotic lactic acid bacteria on obesity and metabolic syndrome. *L. plantarum* KY1032 cell extract reduced fat mass by modulating adipogenesis in maturing preadipocytes [31]. *L. paracasei* decreased fat storage by increasing the level of ANGPTL4 [29]. VSL no. 3, a mixture of bifidobacteria, lactobacilli and *Streptococcus thermophilus*, improved diet-induced obesity, hepatic steatosis and insulin resistance by increasing hepatic natural killer T-cells and reducing inflammatory signaling in mice [39]. On the other hand, it was reported recently that gut microbes play an important role in body weight regulation. Endogenous *Bifidobacterium* spp. were significantly and positively correlated with improved glucose tolerance, glucose-induced insulin secretion and normalized inflammatory tone (decreased endotoxemia, plasma and adipose tissue proinflammatory cytokines) in high-fat-diet and prebiotic-treated mice [40]. Whether supplementation with exogenous probiotic strains has the same mechanism of action is unclear [41]. However, *Lactobacillus* and *Bifidobacteria* are main members of the gut microbiota, and therefore it is worthwhile to investigate the effect of probiotics on the relationship between the gut microbiota and obesity or obesity-related diseases. In summary, the probiotic *L. gasseri* BNR17 lowered body weight and adiposity by increasing the expression of fatty-acid oxidation genes and reducing the levels of leptin and insulin in high-sucrose diet-induced obese mice. This suggests that *L. gasseri* BNR17 may facilitate alleviating metabolic syndrome.
